# Supplementary material for: The Impact of Positive and Adverse Experiences in Adolescence on Health and Wellbeing Outcomes in Early Adulthood
Source: Int J Environ Res Public Health. 2024 Aug 29;21(9):1147. doi: 10.3390/ijerph21091147 (PMC11431561; doi:10.3390/ijerph21091147)
Supplement: Supplementary file 1 [file ijerph-21-01147-s001.zip › ijerph-3142084-supplementary.pdf]

Supplementary Materials

Table S1: Development of the variables used in this study from the data collected and provide by the longitudinal study of Australian youth (LSAY)

		Indicator	Youth age	Measurement items	Responses	Dichotomisation
**Adverse youth experiences/environments**						
Standard of living						AYEs count if
	Living in Poverty	13 items	15	Which of the following possessions do you have?<br>▪ Desk, Own room, study place, computer, software, Internet, calculator, Literature, Poetry, Art, Text books, Dictionary, Dishwasher	Yes/No	Score < 7
	Low money	2 items	16	Are you happy with:<br>▪ 'The money you get each week', 'Your standard of living'	1 = 'Very happy' to 4 = 'Very unhappy'	1 or more unhappy responses
Single parent home						
	Family structure	1 item	15	Which best describes your family?	'Single parent family', 'Nuclear family', 'Mixed family', 'Other', 'Missing'	Single parent family
Experiences of homelessness/transience						
	Not living with parents/relatives	1 item	16 and/or 17	Do you usually live with your parents (or your partner's parents) or somewhere else?	'In your parents home', 'with other relatives', 'In a private house as a boarder', 'in a shared house of flat', 'in a hostel', 'somewhere else' or 'other'	Youth does not live 'In your parents home' or 'with other relatives'
Alienation						

	Not belonging at school	3 items	15	School is a place where:<br>▪ I feel like an outsider, feel awkward, feel lonely	1 = 'Strongly Agree' to 4 = 'Strongly disagree'	2 or more answered agree or strongly agree
	Poor teacher interactions	1 item	15	School is a place where:<br>▪ 'Teachers treat me fairly'	1 = 'Strongly Agree' to 4 = 'Strongly disagree'	Disagree or strongly disagree
Carer responsibilities						
	Looking after other people	1 item	17	Outside study or work, how often do you:<br>▪ Look after people (e.g. young children, grandparents)	Every day to Never	Every day or several times per week.
**Positive youth experiences/environments**						
Good relationships - Trusting and supportive						PYEs count if
	Friendships	1 item	17	How important in your life are:<br>- Your close friends?	On a scale from 0 - 10, where 0 is not at all important and 10 is very important	Score > 7
	Family relationships	1 item	17	How important in your life are:<br>- Family members you live with?		Score > 7
	Supportive adults	4 items	17	How important in your life are:<br>- Relatives you don't live with?<br>- Your Neighbours?<br>- Your friends parents?<br>- Your parents' friends or workmates?		2 x Score > 7
	Positive teacher relationship	2 items<br><br>(alpha = 0.66)	15	'Most of my teachers really listen to what I have to say?', and 'If I need extra help, I will receive it from my teachers?'	1 = 'Strongly Agree' to 4 = 'Strongly disagree'	1 or more answered positively
Supportive neighbourhood and learning environments						

Belonging at school	3 items (alpha = 0.59)	15	School is a place where:<br>I Make friends, Feel I belong, and I think I'm liked	1 = 'Strongly Agree' to 4 = 'Strongly disagree'	2 or more answered positively
Good student-teacher relationships	2 items (alpha= 0.71)	15	'Students get along well with most teachers', and 'Most teachers are interested in students' well-being'	1 = 'Strongly Agree' to 4 = 'Strongly disagree'	1 or more answered positively
School safety	1 item	15	My school is a place where: I feel safe and secure	1 = 'Strongly Agree' to 4 = 'Strongly disagree'	Agree or Strongly agree
Neighbourhood safety	1 item	17	How safe do you feel walking alone after dark in your neighbourhood?	1= 'Very Safe' to 4 = 'Very unsafe'	Safe or Very safe
Social engagement and enjoyment					
Activity: School organised	6 items<br><br>(alpha = 0.5)	15	How often did you take part in the following school-organised activities:<br>- Sport, Music, debating, drama, peer support, school-sponsored volunteer activities?	1 = 'at least once a week, 2 = 'at least once a month', 3 = 'at least every 3 months', 4 = 'at least once per year' and 5 = 'never'	Any activity completed monthly or more
Activity: Non-school	2 items<br><br>(alpha = 0.59)	16 and 17	How often do you: Take part in any community-based activity?		Any activity completed monthly or more
**Outcomes**					
Education, Training and Employment					Outcome if
Employed	1 item	25	Employment status	'FT', 'PT', 'Not working (unemployed or not in labour force)', 'Working, but working time unknown'	Full-time, Part-time or unknown hours

	Highest education level	1 item	25	Highest qualification level completed	9 item list ranging from Certificate I to a Postgraduate degree (PhD/Masters)	Certificate IV (post secondary school trade or equivalent qualification) or above
	Education	1 item	25	Currently in education	‘Full-time’, ‘Part-time’, ‘Unknown’, ‘Not Studying’	FT or PT
Welfare						
	Government payments	1 item	25	Which government payments are you currently receiving?	Full list (inc. Parenting Allowance, Rent Assistance)	Any or None
Health						
	General Health	1 item	25	In general, would you say your health is:	1 = Excellent to 5 = Poor	Good to Excellent
	Mental health	1 item	25	Kessler 6 - In the past 4 weeks, how often did you feel:<br>- Nervous, Hopeless, Restless or Fidgety, Everything is an effort, So sad nothing would cheer you up, Worthless	1 = ‘None of the time’ to 5 = ‘All of the time’. Total scores range from 6 = Good to 30 = Very poor	Total score under 18
